# Increased mucosal neutrophil survival is associated with altered microbiota in HIV infection

**DOI:** 10.1371/journal.ppat.1007672

**Published:** 2019-04-11

**Authors:** Tiffany Hensley-McBain, Michael C. Wu, Jennifer A. Manuzak, Ryan K. Cheu, Andrew Gustin, Connor B. Driscoll, Alexander S. Zevin, Charlene J. Miller, Ernesto Coronado, Elise Smith, Jean Chang, Michael Gale, Ma Somsouk, Adam D. Burgener, Peter W. Hunt, Thomas J. Hope, Ann C. Collier, Nichole R. Klatt

**Affiliations:** 1 Department of Pharmaceutics, University of Washington, Seattle, WA, United States of America; 2 Washington National Primate Research Center, Seattle, WA, United States of America; 3 Biostatistics and Biomathematics Program, Public Health Sciences Division, Fred Hutchinson Cancer Research Center, Seattle, WA, United States of America; 4 Department of Pediatrics, Miller School of Medicine, University of Miami, Miami, FL, United States of America; 5 Center for Innate Immunity and Immune Disease, Department of Immunology, University of Washington School of Medicine, Seattle, WA, United States of America; 6 Division of Gastroenterology, University of California, San Francisco, San Francisco, CA, United States of America; 7 National HIV and Retrovirology Labs, Public Health Agency of Canada, Winnipeg, Manitoba, Canada; 8 Departments of Obstetrics & Gynecology and Medical Microbiology, University of Manitoba, Winnipeg, Manitoba, Canada; 9 Unit of Infectious Diseases, Department of Medicine Solna, Center for Molecular Medicine, Karolinska Institute, Stockholm, Sweden; 10 Division of Experimental Medicine, University of California, San Francisco, San Francisco, CA, United States of America; 11 Department of Cellular and Molecular Biology, Feinberg School of Medicine, Northwestern University, Chicago, IL, United States of America; 12 Department of Medicine, University of Washington, Seattle, WA, United States of America; Vaccine Research Center, UNITED STATES

## Abstract

Gastrointestinal (GI) mucosal dysfunction predicts and likely contributes to non-infectious comorbidities and mortality in HIV infection and persists despite antiretroviral therapy. However, the mechanisms underlying this dysfunction remain incompletely understood. Neutrophils are important for containment of pathogens but can also contribute to tissue damage due to their release of reactive oxygen species and other potentially harmful effector molecules. Here we used a flow cytometry approach to investigate increased neutrophil lifespan as a mechanism for GI neutrophil accumulation in chronic, treated HIV infection and a potential role for gastrointestinal dysbiosis. We report that increased neutrophil survival contributes to neutrophil accumulation in colorectal biopsy tissue, thus implicating neutrophil lifespan as a new therapeutic target for mucosal inflammation in HIV infection. Additionally, we characterized the intestinal microbiome of colorectal biopsies using 16S rRNA sequencing. We found that a reduced *Lactobacillus*: *Prevotella* ratio associated with neutrophil survival, suggesting that intestinal bacteria may contribute to GI neutrophil accumulation in treated HIV infection. Finally, we provide evidence that *Lactobacillus* species uniquely decrease neutrophil survival and neutrophil frequency *in vitro*, which could have important therapeutic implications for reducing neutrophil-driven inflammation in HIV and other chronic inflammatory conditions.

## Author summary

HIV infection results in chronic immune activation that leads to increased risk of other diseases and premature death, and this has been linked to gastrointestinal tract (GI) damage in infected individuals. In this study, we investigated neutrophils, a cell involved in the immune response to pathogens, in colorectal tissue of HIV-infected individuals receiving treatment. Because neutrophils use methods to contain pathogens that can also damage tissue and have been implicated in tissue damage in other GI diseases, it has been proposed that they contribute to GI damage in HIV infection. However, the role of neutrophils in GI damage in HIV has not been well studied. This study quantifies neutrophils in relation to other white blood cells in the GI tissues in HIV infection and demonstrates that they are increased in the GI in infected individuals. Additionally, we present evidence that neutrophils in the GI in HIV-infected individuals have a longer lifespan, which represents one potential reason for their increased frequency. Finally, we present data that different bacteria that naturally reside in the GI can alter neutrophil lifespan and that changes in the relative abundances of these bacteria in HIV infection may be contributing to increased neutrophil lifespan.

## Introduction

Gastrointestinal (GI) mucosal damage and immune dysfunction drive chronic inflammation and microbial translocation in HIV infection, which predict and likely contribute to non-infectious comorbidities and mortality[1–5]. Although long-term antiretroviral therapy (ART) partially restores mucosal damage, a degree of mucosal immune dysfunction and inflammation persists and is associated with morbidities and mortality[6–8]. Improving the understanding of this persistent mucosal dysfunction and inflammation during ART is a major hurdle for the development of targeted therapies that may promote health and decrease morbidities and mortality in HIV-infected individuals.

Neutrophils, the most abundant immune cell, are the first responders to most infections and are crucial in the immune response to bacterial and fungal pathogens[9]. However, the role of neutrophils in HIV infection is not well understood. Imaging studies assessing myeloperoxidase (MPO), an enzyme produced and secreted by neutrophils, suggest that neutrophils accumulate in the GI tract in treated and untreated HIV infection, yet the frequency and functionality of accumulated neutrophils has yet to be examined[10]. A better understanding of the mechanisms involved in neutrophil accumulation in the GI in HIV infection is necessary to develop new strategies to alleviate GI inflammation in HIV infection.

While neutrophils are critical in protection from infections, aberrant neutrophil responses can also be harmful. Neutrophil infiltration in the colonic mucosa is one of the distinguishing characteristics of acute inflammation present in inflammatory bowel disease and correlates with disease severity[11–13]. Neutrophil lifespan is tightly regulated in order to limit unintended damage to tissues by secreted reactive oxygen species and granular enzymes meant to degrade extracellular matrix and disrupt tight junctions[14]. Increased neutrophil lifespan is observed under inflammatory conditions and has been attributed to both direct interaction with microbes and the release of cytokines from other immune cells[15] [16,17].

In HIV-infected individuals, studies have reported that the delicate balance of healthy bacterial communities is perturbed, resulting in microbial dysbiosis [18–22]. Importantly, dysbiosis remained evident in individuals on ART and associated with disease progression [19]. However, recent studies demonstrated that these previous results were likely confounded by sexual orientation, for which the study designs did not adequately match or control [23,24]. Taking this into account, another recent study matched controls based on sexual orientation and found both men who have sex with men (MSM)-specific alterations and HIV-specific alterations in MSM and women[25]. Therefore, it is evident that some combination of infection and sexual orientation alters the microbiome in infected individuals. However, to what degree infection itself and lifestyle parameters contribute to microbial alterations in infected individuals requires further study. Additionally, a recent study of experimental dysbiosis induced by antibiotics in rhesus macaques did not lead to increased disease progression in untreated SIV-infected animals, suggesting that dysbiosis may be co-associated with disease progression rather than causative [26]. However, further studies are required in HIV-infected and uninfected human populations to determine the contributions of microbiome dysbiosis in the context of untreated and treated HIV infection.

The effects of microbiome alterations on intestinal neutrophils have not been previously assessed, and given that Toll-like receptor (TLR) activation and cytokine stimulation regulate neutrophil survival, changes in the microbiome composition could alter neutrophil lifespan. In this study, we hypothesized that HIV-infected individuals would have increased neutrophil frequencies in the lower GI with reduced neutrophil apoptosis. We further hypothesized that different bacterial species would differentially contribute to alterations in neutrophil apoptosis.

## Results

### Increased neutrophil frequencies in colorectal biopsies of HIV-infected, ART-suppressed individuals

Previous studies reporting neutrophil infiltration in HIV infection and the nonhuman primate model of SIV have relied solely on MPO staining of neutrophils measured via microscopy[10,27]. Because MPO secretion is increased upon neutrophil activation, it is unclear if increased MPO in the tissues represents increased neutrophil frequency or increased neutrophil activation. In addition, in some cases of inflammation, tissue macrophages also produce MPO and stain positive for the enzyme[28]. For these reasons, we developed a multicolor flow cytometry-based approach that distinguishes neutrophils in the context of other leukocytes in order to obtain a more quantitative measure of neutrophil frequencies in the GI during HIV infection. Using this panel, we were able to identify neutrophils in blood (Supplementary Fig 1A in S1 Supporting Information) and fresh GI tissue (Supplementary Fig 1B in S1 Supporting Information) to assess the frequency of neutrophils as the percentage of total live CD45+ cells.

**Fig 1:**
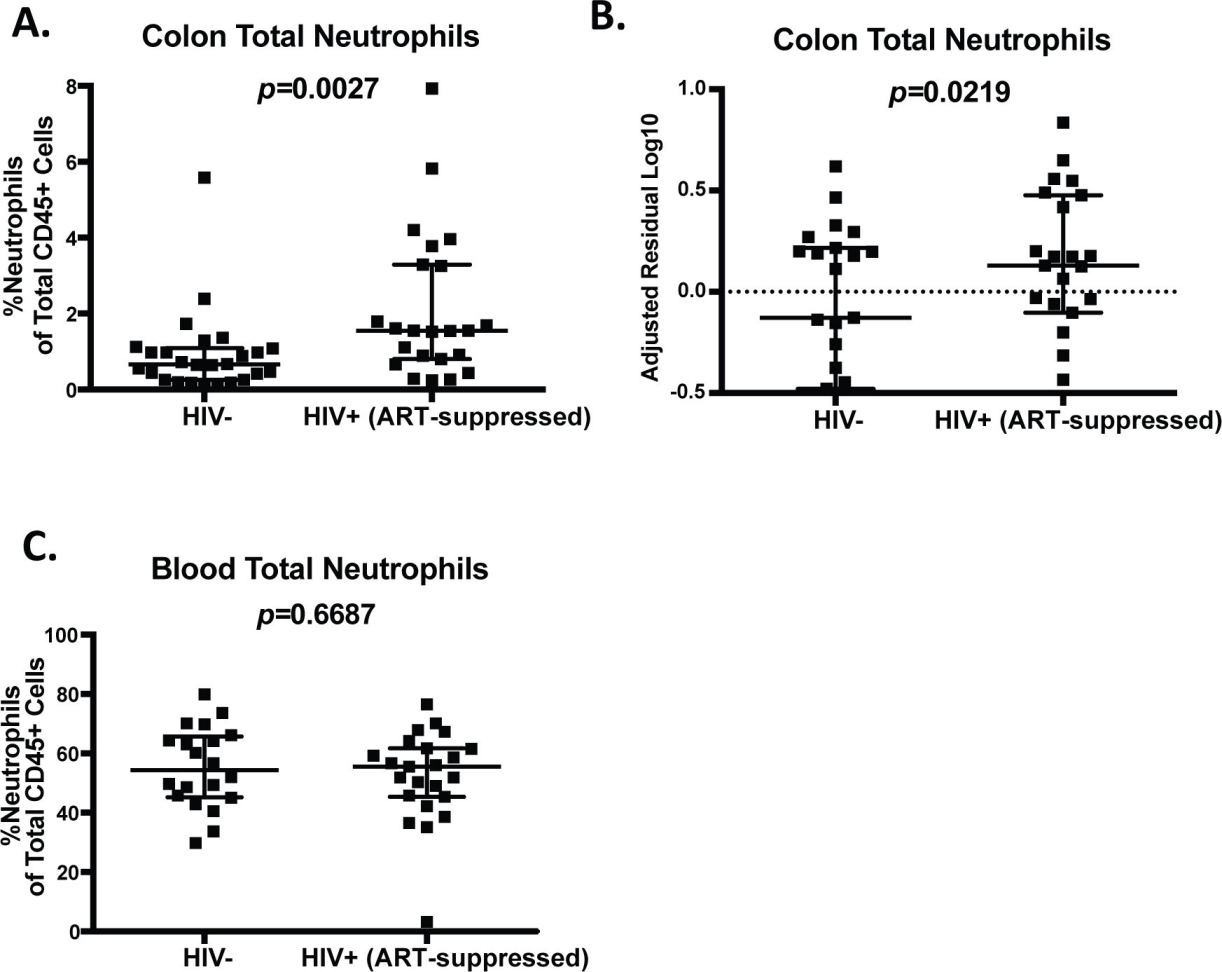
Increased gastrointestinal neutrophil frequency in HIV-infected, ART-suppressed individuals. A.) The frequency of neutrophils as a percentage of total leukocytes isolated from colorectal biopsies of HIV-infected, ART-suppressed individuals and uninfected controls as measured by flow cytometry. B.) Depiction of the log10 residuals of data presented in (A) from a multivariate analysis adjusted for age, race, sex, and sexual orientation. C.) The frequency of neutrophils as a percentage of total leukocytes in whole blood from HIV-infected, ART-suppressed individuals and uninfected controls as measured by flow cytometry. Statistical differences in neutrophil frequencies between infected and uninfected individuals (A and C) were determined by Mann-Whitney test and a multivariate regression analysis (B) with a p-value<0.05 considered significant.

Colorectal biopsies from a total of 40 HIV-infected, ART-suppressed individuals and 35 HIV-uninfected individuals were collected for this study (Supplementary Fig 2 in S1 Supporting Information). Of these colorectal biopsy samples, neutrophil frequencies were assessed by flow cytometry in real-time in 23 HIV-infected, ART-suppressed and 25 HIV-uninfected participants. Table 1 describes relevant participant demographic information including age, sex, sexual orientation, race/ethnicity, CD4+ T cell count, and time since HIV diagnosis for this subset of individuals. We found increased neutrophil frequencies in the GI of HIV+ individuals compared to uninfected controls (Fig 1A). Importantly, this increase remained when assessed using a multivariate analysis adjusting for age, race, sex, and sexual orientation (Fig 1B). This increase in neutrophils is specific to the GI tract, as we observed no increase in neutrophil frequency in the blood of HIV-infected, ART-suppressed individuals (Fig 1C). These data are the first to demonstrate neutrophils are increased in the GI tract relative to other leukocytes in HIV-infected individuals despite suppressive ART.

**Table 1:** Study participant demographics.

	HIV+ (ART-Suppressed) n=23	HIV- n=25
**Sex, No. (%)**		
Female	4 (17.4%)	8 (32.0%)
Total Male	19 (82.6%)	17 (68.0%)
Male, MSM	19 (100.0%)	6 (35.3%)
Male, Non-MSM	0 (0.0%)	11 (64.7%)
**Age, Median (IQR)**	52 (48-60)	52.5 (48-60)
**Race/Ethnicity, No. (%)**		
Unknown	1 (4.3%)	2 (8.0%)
White, non-Hispanic or Latino	15 (65.2%)	13 (52.0%)
Black or African American	5 (21.7%)	6 (24.0%)
Latino	0 (0.0%)	1 (4.0%)
Native American/Alaska Native	2 (8.7%)	2 (8.0%)
Asian	0 (0.0%)	1 (4.0%)
**Years since HIV diagnosis, Median (IQR)**	17.25 (8.9-25.1)	--
**CD4+ T cell Count, Median (IQR)**	699 (524.5-838.5)	--

### Increased neutrophil survival in colorectal biopsies of HIV-infected, ART-suppressed individuals

**Fig 2:**
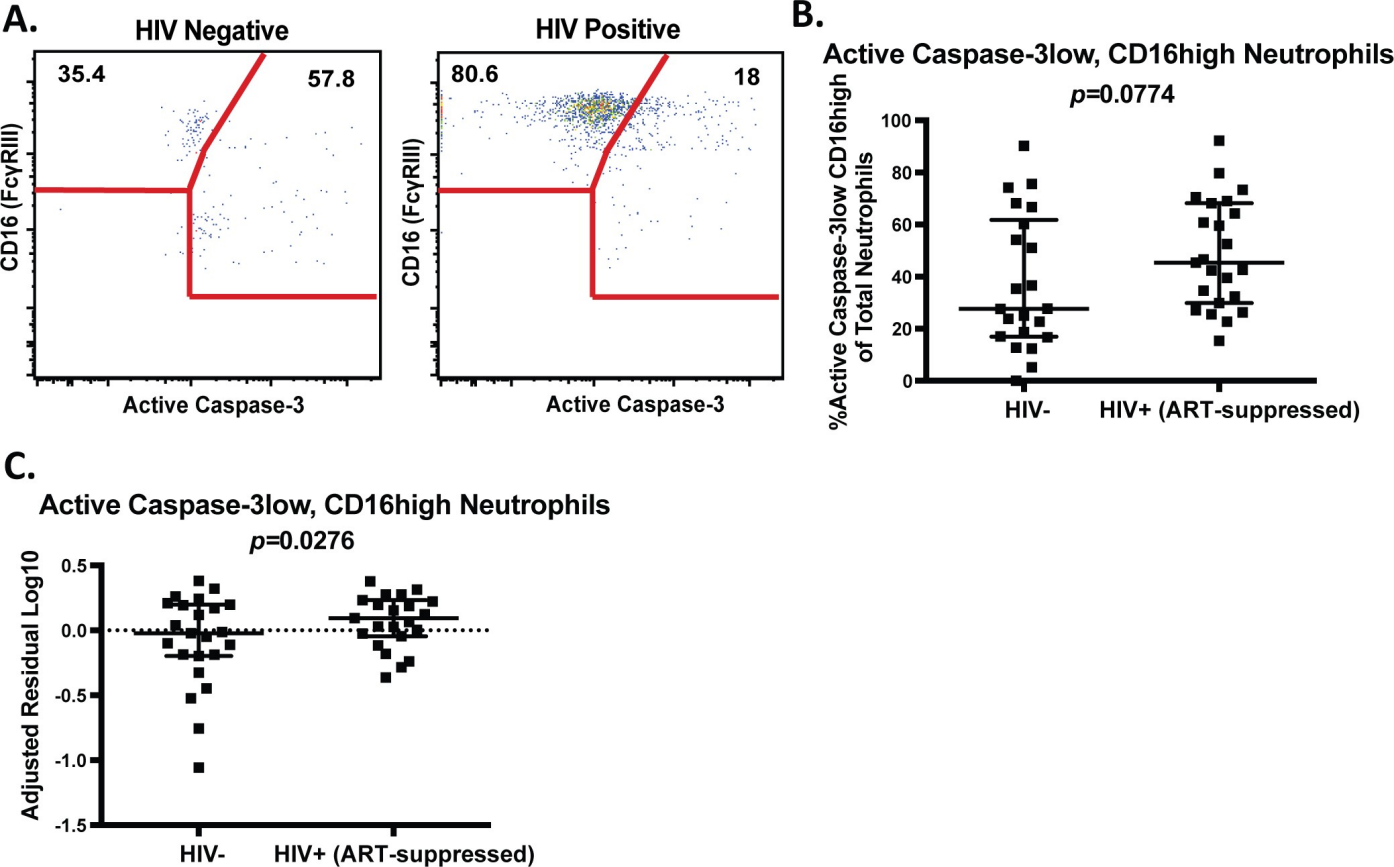
Increased gastrointestinal neutrophil survival in HIV-infected, ART-suppressed individuals A.) Example gating of active Caspase-3 low, CD16 high neutrophils isolated form colorectal biopsies. B.) The frequency of total neutrophils expressing low levels of active Caspase-3 and high levels of CD16 in colorectal biopsies of HIV-infected, ART-suppressed individuals and uninfected controls as measured by flow cytometry. C.) Depiction of the log10 residuals of data presented in (B) from a multivariate analysis adjusted for age, race, sex, and sexual orientation. Statistical differences in neutrophil frequencies between infected and uninfected individuals (B) were determined by Mann-Whitney test and a multivariate regression analysis (C) with a p-value<0.05 considered significant.

One potential mechanism for elevated frequencies of neutrophils in the colon in HIV is prolonged neutrophil survival. Neutrophil clearance is an important mechanism for tissue homeostasis, and neutrophils are generally short-lived, with findings from studies investigating neutrophil lifespan *in vivo* ranging from 8 hours to 5 days[29]. The least inflammatory mechanism of neutrophil clearance from tissues is caspase-3 mediated apoptosis followed by engulfment by macrophages[30–32]. Neutrophils undergoing apoptosis demonstrate reduced surface CD16 expression[33] and those with reduced CD16 expression also demonstrate reduced functionality[34]. Therefore, in order to evaluate neutrophil survival, we measured non-apoptotic, functional neutrophils as those expressing high levels of CD16 and low levels of active Caspase-3 in leukocytes isolated from colorectal biopsies (Fig 2A). Indeed, we found that the frequency of these surviving, functional neutrophils was increased in biopsies from HIV+ individuals compared to uninfected controls (Fig 2B), which was significant in a multivariate analysis adjusted for age, race, sex, and sexual orientation (Fig 2C). While neutrophil infiltration in inflammatory bowel disease has been attributed in part to delayed neutrophil apoptosis[35], these are the first data demonstrating this as a potential mechanism in ongoing intestinal inflammation in HIV infection. Importantly, we observed no differences in total neutrophils or neutrophil lifespan based on sex or sexual orientation in both an unadjusted analysis and after adjustment for HIV status (Supplementary Fig 3 in S1 Supporting Information).

### Mucosal microbiome composition of colorectal biopsies associates with HIV status

**Fig 3:**
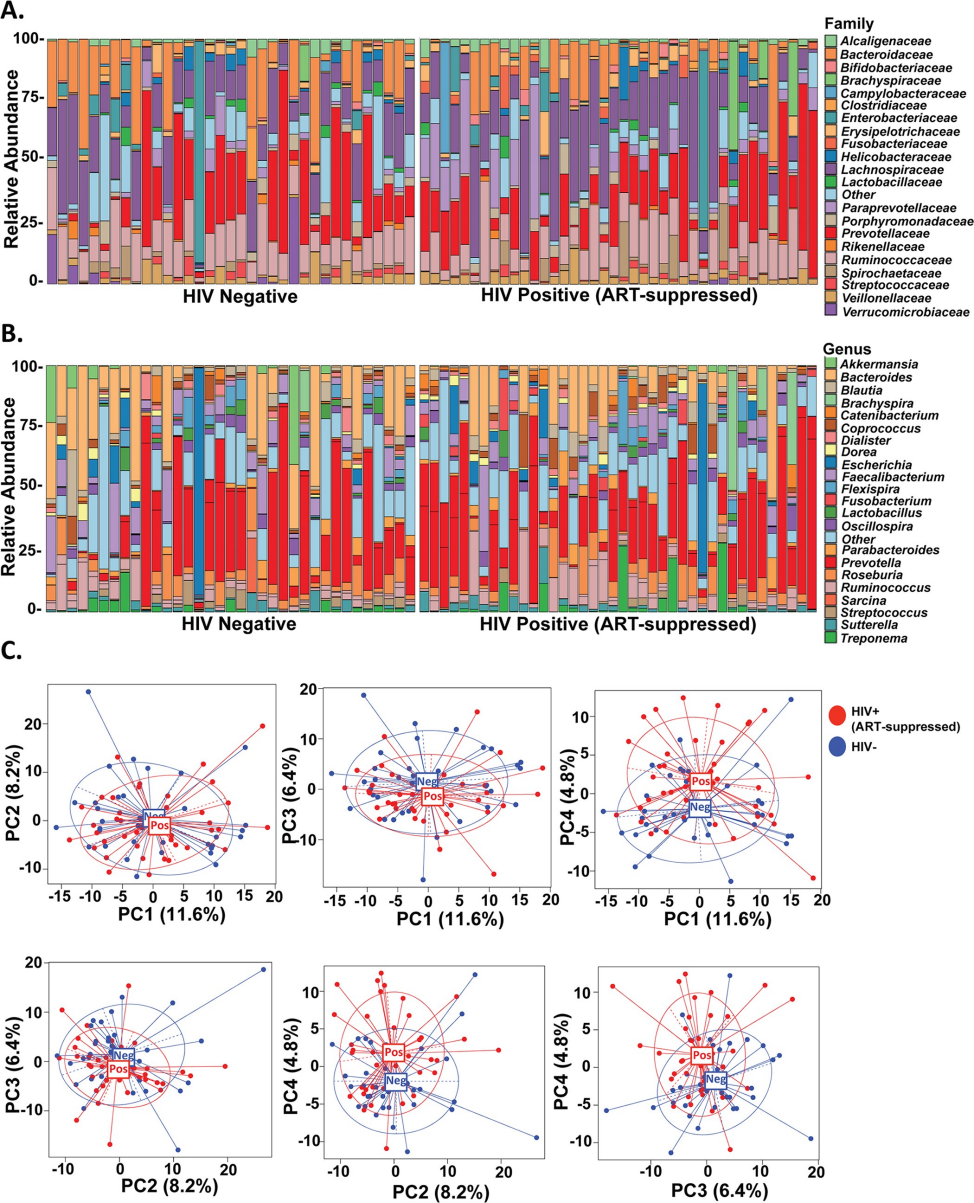
Microbial composition of colorectal biopsies associates with HIV status. Microbial composition was measured by 16S rRNA sequencing from colorectal biopsies obtained from HIV-negative and HIV-positive, ART-suppressed individuals. A.) Relative abundances of the 21 most abundant families with the remaining taxa categorized as “Other”. B.) Relative abundance of the 22 most abundant genera with the remaining taxa categorized as “Other”. C.) Principal component plots visualizing differences in microbial composition between HIV-negative and HIV-positive, ART-suppressed individuals following adjustment for age, race, sex, and sexual orientation.

Alterations to the intestinal microbiome in HIV-infected individuals are well-documented[36]. Given the extensive evidence that microbes and microbial ligands impact neutrophil survival through both direct interactions and by influencing cytokine release by other immune cells[15,16], we sought to determine whether alterations in microbial composition associated with differential neutrophil frequency, function and survival. To do this, we assessed the mucosal microbiome composition by 16S rRNA gene sequencing of colorectal biopsies collected from all 40 HIV-infected, ART-suppressed individuals and all 35 HIV-uninfected individuals. We focused on bacterial composition at both the family and genus taxonomic levels (Fig 3A and 3B) and observed a modest significant association between overall microbial composition at the genus level and HIV status when HIV-infected, ART-suppressed individuals were compared to uninfected controls (*p*=0.041 as determined by MiRKAT analysis). Importantly, this association remained when adjusted for age, race, sex, and sexual orientation, as can be visualized by principle component analyses of the adjusted relative abundances (*p*=0.035, Fig 3C). Few individual genera significantly associated with HIV status in either the unadjusted (Supplementary Fig 4A in S1 Supporting Information) or adjusted analyses (Supplementary Fig 4B in S1 Supporting Information) when the false discovery rate was taken into account (q-value<0.05), which may be due to low sample size.

### Mucosal microbiome composition of colorectal biopsies associates with sexual orientation in men

**Fig 4:**
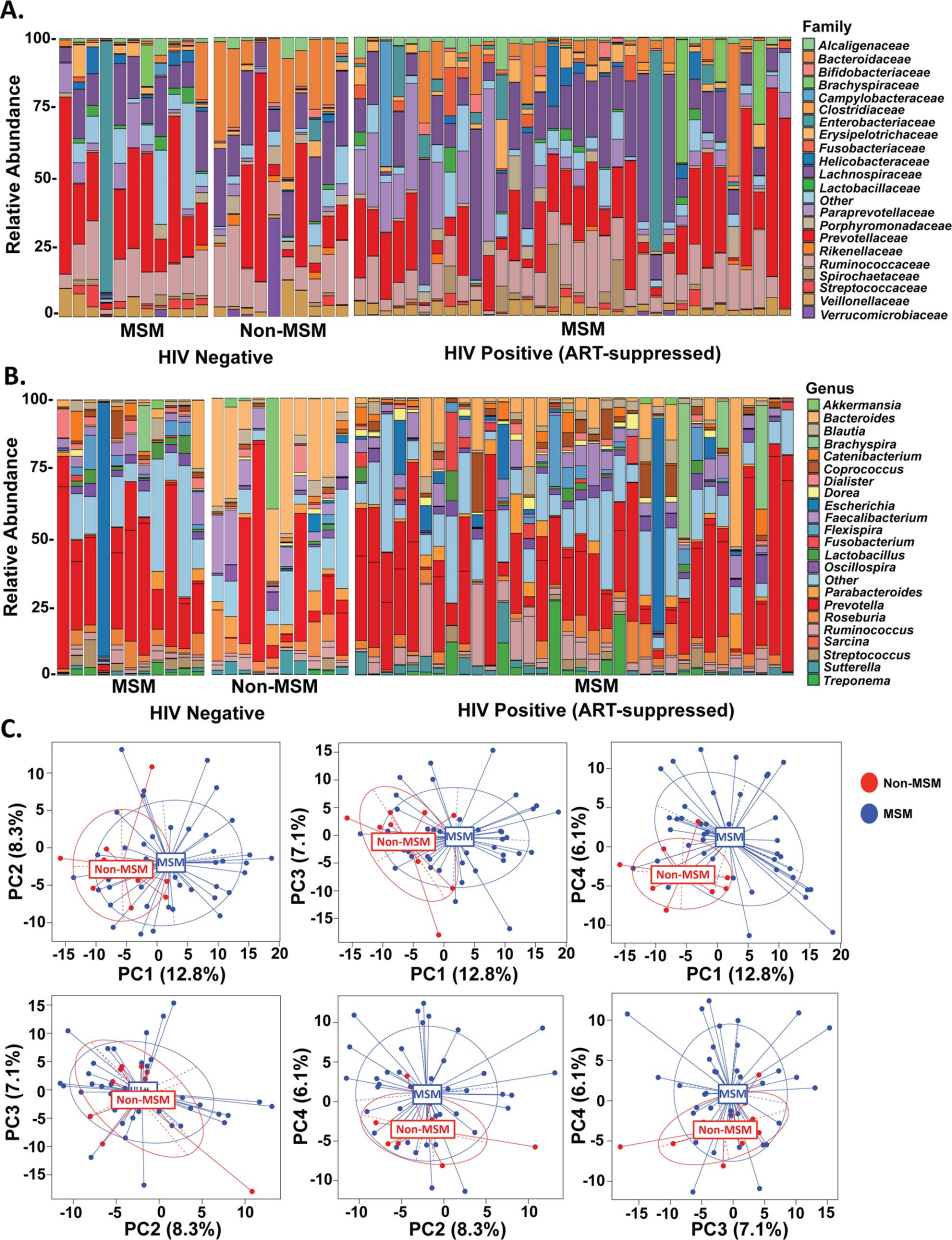
Microbial composition of colorectal biopsies associates with sexual orientation in men. Microbial composition of colorectal biopsies from HIV-infected, ART-suppressed and uninfected men as measured by 16S rRNA sequencing and categorized as MSM or non-MSM. A.) Relative abundances of the 21 most abundant families with the remaining taxa categorized as “Other”. B.) Relative abundance of the 22 most abundant genera with the remaining taxa categorized as “Other”. C.) Principal component plots visualizing differences in microbial composition between MSM and non-MSM individuals following adjustment for age, race, sex, and HIV-status.

Importantly, recent studies have demonstrated that the dysbiosis previously attributed to HIV infection may actually be a result of sexual risk behaviors, as men who have sex with men (MSM) had an increased abundance of *Prevotella*, independent of HIV status [23,25]. This highlights the importance of investigating confounding demographic factors in comparisons of HIV infected and uninfected populations. Therefore, we further investigated the microbial composition of the men in this cohort based on sexual orientation at both the family and genus taxonomic levels (Fig 4A and Fig 4B). We observed a significant association between microbial composition at the genus level and sexual orientation as categorized by MSM or non-MSM (*p*=0.002, Fig 4C as determined by MiRKAT analysis). This association remained when adjusted for age, race, and HIV status (*p*=0.020). Several alterations in bacterial taxa were associated with sexual orientation in both the adjusted and unadjusted analyses, including a loss of bacteria in the *Bacteroides* genus and the *Barnesiellaceae* family and an increase in bacteria of the *Streptococcus* genus (Supplementary Fig 5 in S1 Supporting Information). The *Prevotella* genus only significantly associated with sexual orientation prior to adjustment for HIV status.

### *Lactobacillus*:*Prevotella* ratio associates with GI Neutrophil survival *in vivo*

In order to examine the potential impact of bacterial composition on neutrophils, we assessed associations between all taxa found in at least 25% of individuals and colorectal neutrophils. We found no associations between individual taxa and total neutrophils or neutrophil survival in an unadjusted analysis or following adjustment for age, race, sex, sexual orientation, and HIV status once the false discovery rate threshold was applied (S2 Supporting Data). It is possible that our sample size is too small to detect significant associations across all taxa when adjusting for multiple comparisons. Therefore, we next focused on individual bacteria that we hypothesized could impact neutrophil survival, particularly in HIV-infected populations.

**Fig 5:**
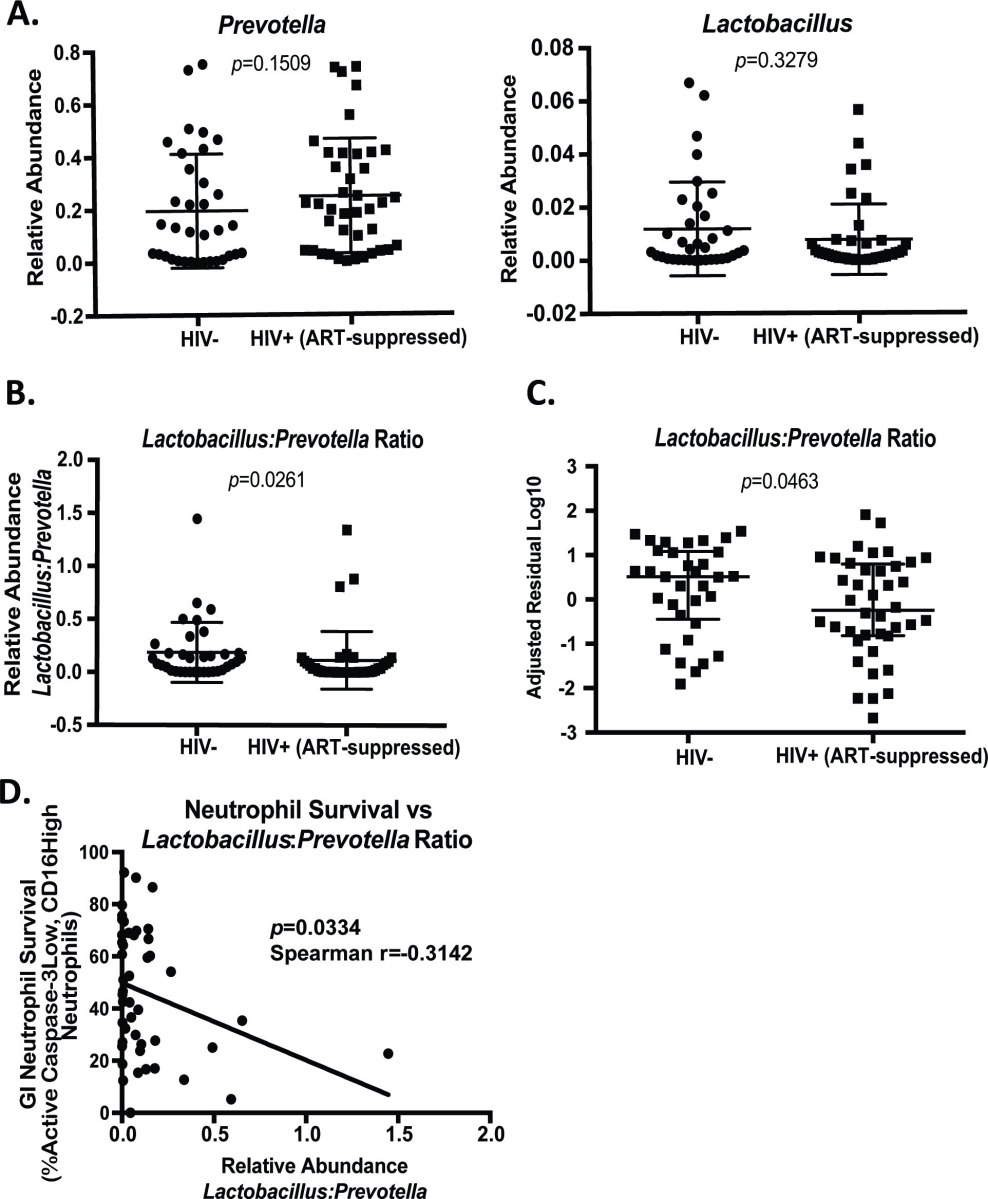
Lactobacillus:Prevotella ratio associates with GI Neutrophil survival in vivo. A.) Relative abundances of Prevotella and Lactobacillus genera in HIV-negative and HIV-positive, ART-suppressed individuals as measured by 16S rRNA sequencing of colorectal biopsies. B.) Ratio of Lactobacillus:Prevotella (relative abundances) in HIV-negative and HIV-positive, ART-suppressed individuals. C.) Depiction of the log10 residuals from data presented in (B) from a multivariate analysis adjusted for age, race, sex, and sexual orientation. D.) Correlation of Lactobacillus:Prevotella ratio and GI neutrophil survival as measured by the percentage of active Caspase-3 low, CD16 high neutrophils isolated from colorectal biopsies of HIV-negative and HIV-positive, ART-suppressed individuals. Statistical differences between infected and uninfected individuals (A and B) were assessed by Mann-Whitney test and a multivariate regression analysis (C) with a p-value<0.05 considered significant. Correlation assessed by Spearman rank correlation test.

Specific bacteria altered in certain HIV-infected populations have been shown to impact mucosal immune cells [18,37]. Of note, the *Prevotella* genus had the highest average relative abundance in our study population, and increased *Prevotella* in one HIV-infected population associated with increased mucosal T cell and dendritic cell activation [18]. In a follow-up study, the authors further reported the ability of *Prevotella* species to activate leukocytes *in vitro* by demonstrating that myeloid dendritic cells stimulated with *Prevotella stercorea* and *Prevotella copri* produced increased cyctokine[38]. Although the authors did not take into account sexual orientation in these studies, another recent study correlated *Prevotella* with T cell activation within an MSM population[25]. It remains unclear if the link between *Prevotella* and immune activation can be applied to other populations of HIV-infected individuals. It has been particularly difficult to assess these associations in non-MSM, HIV-infected men due to the insufficient number of available samples [25], which unfortunately were also unavailable for our study. However, given *in vivo* and *in vitro* evidence that *Prevotella* species are able to activate leukocytes, we hypothesized that *Prevotella* could impact neutrophil survival. *In vitro* assessment using a reporter cell line demonstrated that *Prevotella copri* activated NF-kb through a TLR-4 dependent mechanism [39] and NF-kb activation is known to drive survival factors in neutrophils[40]. Additionally, LPS has been shown to increase neutrophil survival both through direct TLR-4 activation on neutrophils and by inducing the release of TNF-α and IL-1β from monocytes[15]. Contrarily, reductions in *Lactobacillus* have been shown to impact gut health and immune function [21,41], and *Lactobacillus* species have been demonstrated to induce apoptosis in epithelial cells and myeloid cells[42]. Therefore, we hypothesized that alterations in these genera may impact neutrophil survival in the GI of HIV-infected, ART-suppressed individuals, and we sought to assess the relationships between these bacteria and neutrophils. While we observed no significant differences in the relative abundances of bacteria in the *Prevotella* or *Lactobacillus* genera (Fig 5A), we observed a significant difference in the ratio of *Lactobacillus*:*Prevotella* between HIV-infected, ART-suppressed and uninfected individuals, suggesting an altered balance of these genera (Fig 5B). Additionally, the *Lactobacillus*:*Prevotella* ratio remained significantly altered following adjustment for age, race, sex, and sexual orientation (Fig 5C). Importantly, the *Lactobacillus*:*Prevotella* ratio correlated with neutrophil survival in leukocytes isolated from colorectal biopsies from the same individual (Fig 5D), suggesting that an alteration in bacterial composition may impact neutrophil survival *in vivo*.

Given the recent interest in balance analyses of microbiome data, we performed a *selbal* analysis to determine the microbial signature predictive of increased neutrophil lifespan (Supplementary Fig 6 in S1 Supporting Information). The balance we identified as most closely associated with neutrophil lifespan included three genera in the numerator and ten genera in the denominator groups. Interestingly, *Prevotella* was among the bacteria in the numerator group, which includes bacteria that may be positively contributing to increased neutrophil lifespan. Additionally, *Lactobacillus* was among the bacteria in the denominator group, or those that may negatively affect neutrophil lifespan. These results suggest that these bacteria may have a role within global microbiome composition changes in altering neutrophil lifespan. However, these analyses should be interpreted with caution given that this method selects bacteria without applying statistical inference. Further study is needed to confirm these relationships *in vivo* with a larger sample size and *in vitro* as culturing methods and commercial strains become available.

### Bacteria differentially affect neutrophil survival *in vitro*

Given our observation that HIV-infected individuals in our study had significantly altered bacterial community composition *in vivo* and previously published data suggesting that bacterial ligands can impact neutrophil lifespan, we examined the ability of bacterial ligands and various whole bacteria to impact neutrophil survival in an *in vitro* culture system with whole blood. These experiments were done using whole blood samples from both HIV-infected and uninfected individuals in order to assess the potential impact of confounding HIV infection on the ability of bacteria to alter neutrophils. In accordance with previous studies, we observed a significantly increased frequency of surviving neutrophils in whole blood after a 20-hour incubation with TLR-4 and TLR-2 agonists compared to unstimulated controls (Supplementary Fig 7A in S1 Supporting Information). Isolated neutrophils incubated with TLR-4 and TLR-2 agonists also demonstrated an increased frequency of surviving neutrophils after a 20-hour incubation that did not reach statistical significance, and to a much lesser extent than that observed in whole blood (Supplementary Fig 7B in S1 Supporting Information). These data suggest that both direct interactions with neutrophils and soluble factors released by other leukocytes impact neutrophil survival in the presence of microbial antigens.

**Fig 6:**
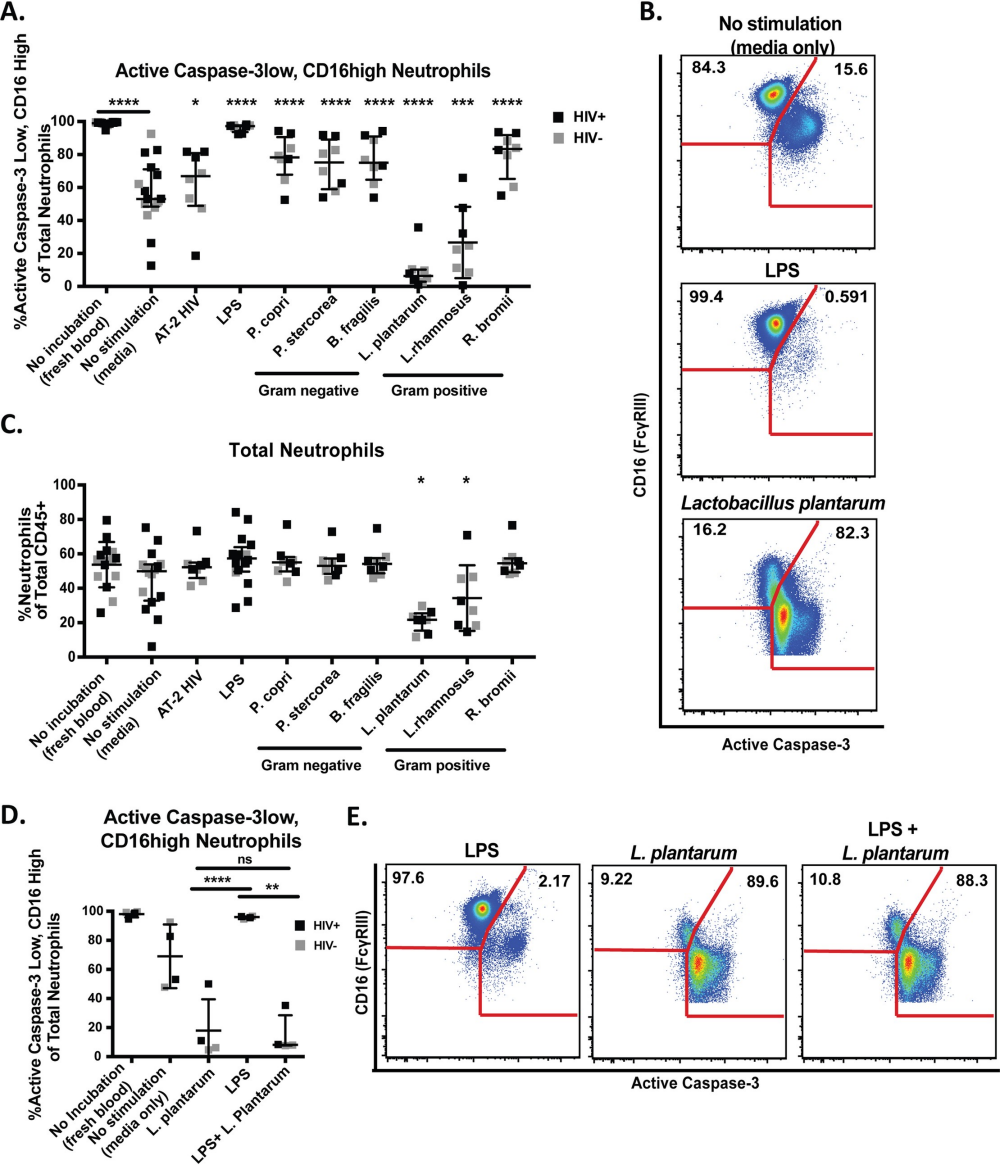
Mucosal bacteria differentially affect neutrophil lifespan. A.) Percentage of active Caspase-3low, CD16high neutrophils in whole blood incubated with different HIV altered mucosal bacteria as measured by flow cytometry. B.)Example staining of active Caspase-3low, CD16high neutrophils after stimulation with LPS and Lactobacillus plantarum. C). Total neutrophil frequencies as a percentage of total CD45+ leukocytes in whole blood incubated with different HIV altered mucosal bacteria. D.) Percentage of active Caspase-3low, CD16high neutrophils in whole blood incubated with LPS alone, Lactobacillus plantarum alone, and LPS with L. plantarum as measured by flow cytometry. E.) Example staining of active Caspase-3low, CD16high neutrophils after stimulation with LPS alone, Lactobacillus plantarum alone, and LPS with L. plantarum. Statistical significance was determined using a paired one-way ANOVA followed by a Dunnett’s post-hoc analysis for multiple comparisons comparing each group to the media control. Asterisks (*) indicate significance by adjusted p-value from post-hoc analysis (*p <0.05, ** p <0.01, *** p <0.001, ****p<0.0001). The statistical difference between fresh blood and blood incubated in media alone was assessed using a Wilcoxon signed-ranked test (****p<0.0001).

We next examined the ability of various bacteria previously reported to be altered in HIV-infected individuals [37] to impact neutrophil survival *in vitro*. All bacterial species significantly increased neutrophil survival after incubation with whole blood, with the exception of *Lactobacillus* species (Fig 6A and Fig 6B). LPS purified from *Escherichia coli* (*E*. *coli)* was used as a positive control due to its ability to significantly impact neutrophil survival in the previous experiment. Importantly, *Lactobacillus plantarum* and *Lactobacillus rhamnosus* decreased neutrophil survival. Additionally, we found that both *Lactobacillus* species decreased total neutrophil frequencies and that the increased neutrophil survival observed upon stimulation by non-*Lactobacillus* bacteria resulted in sustained neutrophil frequencies (Fig 6C). This suggests that the non-*Lactobacillus* bacteria shown to reduce active Caspase-3 expression leads to increased neutrophil survival, rather than causing a different form of cell death such as necrosis. In these experiments, the whole blood neutrophils did not respond differently to any stimulation condition based on HIV status. Additionally, given that these were blood neutrophils, we did not expect other demographic factors such as sexual orientation to have an impact and therefore did not collect such data on these individuals. Also, because *Ruminococcus bromii* and *Bacteroides fragilis* increased neutrophil survival similarly to the *Prevotella* species, we assessed the *Lactobacillus*:*Ruminococcus* and *Lactobacillus*:*Bacteroides* ratios *in vivo* and found no differences based on HIV status and no association with neutrophil survival (Supplementary Fig 8 in S1 Supporting Information).

Given that bacteria *in vivo* are unlikely to be spatially separated and would therefore interact with cells simultaneously, we assessed the ability of *Lactobacillus* to reduce neutrophil survival in the presence of LPS in a subset of individuals. We used the same LPS purified from *E*. *coli* as was used in previous experiments to test the ability of *Lactobacillus* to override strong signals of neutrophil survival. We observed that *L*. *plantarum* incubated with whole blood in the presence of LPS reduced neutrophil survival similarly to that of *L*. *plantarum* alone (Fig 6D and Fig 6E). *L*. *plantarum* is commonly used in therapeutic studies with probiotics and often found in available probiotic supplements. It has also been investigated alone and in supplements with other bacteria for its ability to reduce inflammation in SIV and HIV[43,44]. Therefore, this observation has important implications for the potential use of *Lactobacillus* to therapeutically target neutrophil survival, as it suggests that *Lactobacillus* found in common probiotics could potentially override survival signals induced by other microbes or microbial molecules in the environment.

We additionally assessed the effect of different enteric bacteria on the survival of isolated neutrophils and observed much lower survival among isolated neutrophils compared to those in whole blood after 20 hours of incubation in media alone (Supplementary Fig 9 in S1 Supporting Information). No significant effects of bacterial stimulation were observed after correction for multiple comparisons. It is likely that the isolation procedure activated apoptosis pathways that could not be reversed by subsequent stimulation. We therefore cannot conclude to what extent the bacteria may alter homeostatic apoptosis of isolated neutrophils without this confounding activation upon isolation. Further studies will be necessary to better determine the effects of other leukocytes in the alteration of neutrophil survival by different bacteria.

## Discussion

Neutrophils have been suggested to contribute to intestinal inflammation in HIV infection, however the causes and consequences of neutrophil accumulation in the intestines during infection are not well understood. Here we demonstrate that neutrophil lifespan is altered in the GI in treated HIV infection and report data suggesting a potential link between the intestinal microbiome and neutrophil accumulation. While numerous studies have linked reduced neutrophil apoptosis to disease severity in IBD, these are the first data suggesting that increased neutrophil lifespan contributes to GI neutrophil accumulation in HIV Infection. Further, we report that mucosal bacteria have differential effects on neutrophil survival *in vivo* and *in vitro*, suggesting that an altered microbiome resulting from a combination of HIV infection and lifestyle, such as sexual orientation, may contribute to neutrophil accumulation and inflammation in the GI through effects on neutrophil apoptosis.

We assessed the alterations to the microbiome in this cohort by 16S rRNA gene sequencing of colon biopsies, and we report that microbial composition was modestly associated with HIV status and robustly associated with sexual orientation in men. Taken together, these data suggest that both HIV infection and sexual orientation may contribute to observed alterations in colon microbial composition in HIV-infected individuals in this study. Interestingly, we observed some alterations in bacterial abundances based on HIV status in the colorectal biopsies that differ from the results of previously published studies in HIV infection. Specifically, decreased *Bradyrhizobium* associated with HIV status in our cohort (although this was not significant using a multiple comparisons approach), while a previous study reported increased *Bradyrhizobium* in the duodenum of HIV-infected individuals[45]. However, this previously reported increase was only observed in individuals with abnormal blood CD4+ T cell counts, suggesting that differences in treatment and disease progression likely contribute to differences in observed microbiome alterations. Additionally, we observed that decreased *Peptoniphilus* associated significantly with HIV status following adjustment for sexual orientation, while a previous study reported an increase in the rectum in ART-treated, HIV-infected individuals[46]. This could be due to differences between colon and rectal microbial composition as well as differences in cohort demographics. For instance, the authors note that *Peptoniphilus* is reduced in the penile microbiota following circumcision and is found in vaginal and genitourinary tract infections[47,48], suggesting that cohorts may have different abundances of this bacteria in the lower GI depending on circumcision rates and genital tract microbial composition. Additionally, we observed a positive association between *Bilophila* and HIV status following adjustment for sexual orientation, while a previous study reported reduced *Bilophila* in the stool in HIV-infected individuals in China[49]. However, the Chinese cohort examined included individuals with prior antibiotic use as well as untreated individuals and they did not control or match for sexual orientation. These differences highlight the variability in reported microbiome alterations that could result from different GI sample types and assessing cohorts from different geographic areas with different antibiotic usage, treatment status, and disease progression.

We further examined *Prevotella* and *Lactobacillus*, two genera we hypothesized may impact neutrophil apoptosis based on previously published studies. In this study, although *Prevotella* was among the top genera that associated with HIV status, this association was not significant by a multiple comparisons approach. Further, after we adjusted for demographics and sexual orientation, *Prevotella* no longer associated with HIV status, supporting recently published studies linking *Prevotella* enrichment to MSM sexual orientation rather than HIV status[23]. In accordance with that study, we observed a significant association between *Prevotella* and MSM in this cohort; however, this association did not remain significant after adjusting for HIV status.

Several studies have indicated that *Lactobacillus* is depleted in HIV-infected individuals[45,50] and a higher abundance of gut Lactobacillales was associated with reduced microbial translocation, increased CD4+ T cells in the periphery and gut, and less immune activation in HIV-infected, ART-treated individuals[51]. In this study, *Lactobacillus* did not specifically associate significantly with HIV status after accounting for multiple comparisons. However, we observed a significant difference between the *Lactobacillus*:*Prevotella* ratios in HIV-infected and uninfected individuals, suggesting an altered balance of these bacteria. Additionally, we observed an association between this ratio and neutrophil survival in matched colon biopsies, providing evidence that microbial composition may impact neutrophil survival *in vivo*. Further, *Prevotella* demonstrated the ability to increase neutrophil survival *in vitro*, although this ability was not specific to *Prevotella*, as the *Rumincoccus* and *Bacteroides* species similarly increased neutrophil survival. However, in combination with the *in vivo* ratio and *selbal* balance analyses indicating that *Prevotella* abundance associates with neutrophil survival, these data provide evidence of a role for *Prevotella* in increased GI neutrophil survival within the context of the greater microbial community. Neutrophil survival and *Prevotella* alterations coexist in several inflammatory conditions, including bacterial vaginosis[52,53], rheumatoid arthritis[54–56], and periodontitis[57,58], suggesting that this link warrants further investigation in larger, matched cohorts of HIV-infected individuals. Finally, the *selbal* balance analysis indicates that other bacteria, including *Roseburia* and *Treponema*, may positively associate with neutrophil lifespan *in vivo*. Future studies should assess the effects of different bacterial balances and combinations on neutrophils to better understand the role of altered bacterial abundances in neutrophil lifespan in inflammatory conditions.

Importantly, what is considered to be a healthy microbiome varies geographically, and it has been argued that this is likely due to dietary differences[59,60]. We did not assess diet in these individuals, which could have impacted our ability to detect differences in *Prevotella* and *Lactobacillus* between the infected and uninfected individuals in this cohort, as both genera have a demonstrated link to diet[61]. Future studies are needed to better understand how such important lifestyle and demographic factors may impact the relationship between the microbiome and neutrophil frequency and lifespan *in vivo*.

Given our observation that HIV-infected individuals in our study population had altered microbial composition, we performed *in vitro* assessments of the effects of various enteric bacteria on neutrophils. These experiments revealed the unique ability of *Lactobacillus* species to increase neutrophil apoptosis, which has not been previously reported and has important implications for potential therapeutic intervention in HIV and other diseases of intestinal inflammation. Importantly, the ability of bacteria to alter neutrophil lifespan *in vitro* was not affected by the HIV status of the individual, suggesting that these interactions could occur in the context of HIV infection but are not specific to HIV-infected individuals. Anti-inflammatory effects of various *Lactobacillus* species are well described, and have been attributed to several factors: 1) the ability of superoxide dismutase secreted by *Lactobacillus* to neutralize reactive oxygen species; 2) the inhibition of the NF-κB pathway leading to a reduction in pro-inflammatory cytokines and chemokines; and 3) the expansion of regulatory T cells [62–64]. Indeed, the increase in neutrophil apoptosis we report in the presence of *Lactobacillus* may be caused by NF-κB inhibition, which is known to drive the production of survival factors in neutrophils and be an important regulator of apoptosis[40]. These data also suggest that the previously reported ability of *Lactobacillus* to reduce intestinal inflammation *in vivo* may be a consequence of increased neutrophil apoptosis and reduced neutrophil accumulation[65] [66,67].

In HIV infection, increased microbial translocation and dysbiosis may also result in increased neutrophil recruitment in addition to increased neutrophil lifespan. As such, IL-8, a potent neutrophil chemokine and a regulator of neutrophil survival, is increased in the colorectal mucosa of HIV infected individuals relative to uninfected controls[68,69]. Additionally, studies investigating neutrophil lifespan have demonstrated that neutrophil apoptosis is regulated by both cytokines released by monocytes and by direct interaction in response to TLR stimulation, and it is likely that both contribute to alterations in neutrophil apoptosis induced by bacteria[15]. The relative contribution of direct interactions with neutrophils and secreted factors from other leukocytes should be further assessed. Finally, bacteria produce molecules *in vivo* that could additionally impact neutrophil apoptosis. For example, short chain fatty acids have been demonstrated to inhibit NF-κB activation and attenuate antimicrobial and inflammatory neutrophil responses to LPS[61]. Therefore, the ability of microbial products to alter neutrophil apoptosis and neutrophil-driven inflammation *in vivo* is an important area of future research.

It is important to point out that this study has several limitations. Due to the requirement that samples be processed and analyzed fresh to assess neutrophils, there were geographic and time constraints that inhibited our ability to recruit more individuals for this study or narrow the focus of our recruitment. As such, we have a relatively small sample size and were unable to recruit controls specifically matched for sexual orientation, age, race, and other demographic characteristics. While we applied the appropriate statistical corrections to account for differences in these characteristics between the HIV-infected and control groups, additional studies are necessary to further assess the relationship between neutrophils and the microbiome in HIV infection *in vivo*, particularly in the context of sexual orientation. Likely due to the relatively small sample size, we were unable to provide evidence that microbiome alterations associate with GI neutrophils within HIV-infected individuals only, and our *in vivo* analyses associating microbiome alterations and mucosal neutrophil lifespan included both HIV-infected and HIV-uninfected individuals given the skewed distribution of the variables within each population. A larger cohort with a wider distribution of data is necessary in order to fully assess the contribution of microbial changes to neutrophil alterations in the context of HIV infection *in vivo*. Additionally, we were unable to assess neutrophils or the neutrophil/microbiome relationship in untreated individuals because most individuals that are aware of their status in the geographic areas of this study are on treatment. However, we believe that these data are clinically relevant given that individuals are now treated immediately upon diagnosis. Future studies in areas where there are more untreated individuals could be beneficial to further assess neutrophils in HIV infection and their role in GI inflammation in HIV.

Neutrophil apoptosis has emerged as a therapeutic target for the resolution of acute and chronic inflammation in the lungs, the intestines, and arthritis but no strategies are approved for use in humans to-date[70]. Here, we provide evidence that this may also be a therapeutic target to reduce intestinal inflammation in HIV-infected individuals by demonstrating increased GI neutrophil survival in HIV infection. Further, the ability of *Lactobacillus* to uniquely reduce neutrophil survival and neutrophil frequency suggests that ongoing studies investigating L*actobacillus*-containing probiotics should additionally assess neutrophil accumulation as a potential mechanism for any observed alterations in intestinal inflammation. Finally, these data lead to new avenues of research whereby commensal bacteria, their surface antigens, and their products should be further assessed for their therapeutic ability to reduce neutrophil accumulation and tissue damage in HIV infection and other inflammatory conditions.

## Materials and methods

### Study participants

HIV+ and HIV- study participants were recruited through either the University of Washington Center for AIDS Research, University of California San Francisco SCOPE cohort, Northwestern University, or the University of Washington AIDS Clinical Trials Unit. Biopsies were obtained by either colonoscopy or rectosigmoidoscopy. Blood samples for bacterial stimulations were collected in EDTA from HIV+ and HIV- participants recruited through the University of Washington Center for AIDS research. All HIV+ participants were on potent combination antiretroviral therapy at time of biopsy or blood draw with no detectable plasma viral load. The appropriate Institutional Review Boards approved all protocols and informed written consent was obtained from all participants. Table 1 describes relevant participant demographics information including age, sex, sexual orientation, ethnicity, CD4+ T cell count, and time since HIV diagnosis for the individuals that had biopsies that could be processed, stained in real-time, and met the threshold criteria (described below) for accurate neutrophil measurements by flow cytometry. Demographic characteristics for additional biopsy samples that did not meet these criteria but were able to be assessed by 16S sequencing are included in Supplementary Table 1 in S1 Supporting Information. Blood samples from additional donors were obtained from the University of Washington AIDS Clinical Trials Unit for *in vitro* experiments (as depicted in Fig 6 and Supplementary Figs 7 and 9 in S1 Supporting Information), and HIV status was the only demographic data made available for those individuals.

### Ethics statement

The appropriate Institutional Review Boards approved all protocols and informed written consent was obtained from all participants. Approval numbers at each institution are as follows: Northwestern University: STU00200953: Rectal Biopsies; University of Washington/Harborview Medical Center: STUDY00002763; University of California, San Francisco: 10-01218. All participants were adults >18 years of age.

### Sample processing

Gut biopsies were enzymatically digested with media (RPMI 1640 with 2.05mM L-glutamate, 100U/ml Penicillin, 100μg/ml Streptomycin [all from GE Healthcare, Logan, UT]) supplemented with Liberase (40 μg/ml, Sigma-Aldrich, St. Louis, MO) and DNAse (4 μg/ml, Sigma-Aldrich) for 1 hour at 37°C with vigorous stirring, ground through a 70-μm cell strainer into a single cell suspension, and then analyzed by flow cytometry.

### Neutrophil isolation

Neutrophils were isolated by lysing the red blood cells in whole blood with ACK lysing buffer (ThermoFisher Scientific) and then labeling the leukocytes with CD15 microbeads (Miltenyi Biotec, Bergisch Gladbach, Germany). The labeled cells were then loaded onto a MACS Column, which was placed into a magnetic MACS Separator (both from Miltenyi Biotec). The retained CD15+ cells were then washed with ice-cold buffer (PBS with 0.5% BSA and 2 mM EDTA). The column was then removed from the separator and the labeled cells were eluted in ice-cold buffer. Cells were counted and used immediately in bacterial or TLR stimulation experiments.

### Flow cytometry

Single cell isolations from biopsies were analyzed by flow cytometry immediately after isolation. Biopsies and blood or isolated neutrophils from the bacteria stimulations were stained using the following surface antigen mouse anti-human antibodies with clone denoted in (), from Becton Dickinson, and Co. (BD) Biosciences (Franklin, NJ) unless otherwise stated: CD45 PE-CF594 (HI30),CD11b APC-Cy7 (ICRF44), CD66b PE (Biolegend, G10F5), CD49d PE/Cy5 (Biolegend, 9F10), CD20 Brilliant Violet 570 (Biolegend, 2H7), CD3 Brilliant Violet 570 (Biolegend, UCHT1), CD16 BV605 (3G8), CD15 BV650 (HI98), and CD14 BV786 (M5E2). Following surface staining, cells were permeabilized using Cytofix/Cytoperm (BD Biosciences). Intracellular active Caspase-3 was stained using a v450-conjugated rabbit anti-human antibody (BD Biosciences, C92-605). Stained samples were fixed in 1% paraformaldehyde and collected on an LSR II (BD Biosciences, La Jolla, California). Analysis was performed in FlowJo (version 9.7.6, Treestar Inc., Ashland, Oregon). Samples with less than 100 events in the neutrophil gate were not included in analyses due to an inability to ensure adequate fluorescence separation of populations and therefore accurate gating of the neutrophil cell population.

### 16s rRNA gene sequencing and analyses

Genomic DNA was extracted from colon tissue biopsies using the QIAamp PowerFecal DNA Kit (QIAGEN, Valencia, CA). DNA for 16S rRNA sequencing was processed following the Earth Microbiome Project protocols (http://press.igsb.anl.gov/earthmicrobiome/protocols-and-standards/16s/) with the following modifications. During the library preparation, each DNA sample was amplified in triplicate using the FailSafe PCR System (Epicentre, WI) and the 515FB-806RB primer pair to generate a 400 bp amplicon from the V4 variable regions of the 16S rRNA gene. The triplicates reactions were pooled, quantified using Qubit dsDNA High Sensitivity Assay Kit (ThermoFisher Scientific, Waltham, MA), and visualized using a LabChip GX (PerkinElmer, MA). Using the concentration of the 400 bp peak, 0.4 ng of each library was pooled into a single sample. The ~400 bp amplicon from the pooled sample was isolated using a BluePippen System (Sage Science, MA), cleaned using AMPure XP Beads (Beckman Coulter, IN) and quantified using the KAPA Library Quantification Kit (KAPA Biosystems, MA). Sequencing was carried out as detailed in the EMP protocol; specifically, 7 pM of the pooled library with 30% PhiX phage as a control was sequenced using a 300-cycle Illumina MiSeq Kit.

16S rRNA gene sequence data was analyzed using the QIIME 2 software package[71]. Sequences were classified using the Naïve Bayes classifier trained on Greengenes 13_8 and binned into operational taxonomic units (OTUs) at 99% sequence similarity[72]. OTUs were then classified using Greengenes 13_8 and converted to relative abundance at the family and genus taxonomic levels for visualization and statistical analyses. Taxonomy plots were created in RStudio Version 1.1.422 using the phyloseq package[73].

### Bacterial preparation and stimulations

*Prevotella stercorea* (DSMZ #18206, Braunschweig Germany), *Prevotella copri* (DSMZ #18205), *Bacteroides fragilis* (ATCC #25285, Manassas, Virginia), and *Ruminicoccus bromii* (ATCC #27255) were all grown in anaerobically in chopped meat broth (Hardy Diagnostics, Santa Maria, CA) supplemented with 1% trace minerals (ATCC), 1% vitamin supplements (ATCC), 0.05% Tween 89 (Sigma-Aldrich, Saint Louis, MO), 29.7 mM acetic acid (Sigma-Aldrich), 8.1 mM proprionic acid (Sigma-Aldrich), and 4.4 mM butyric acid (Sigma-Aldrich). *Acinetobacter junii* (ATCC #17908) was grown aerobically in nutrient agar (BD). *Lactobacillus plantarum* (ATCC #14917) and *Lactobacillus rhamnosus* (ATCC #53103) were grown aerobically in MRS broth (BD). All bacteria were counted using CountBright Absolute Counting Beads (Thermo Fisher Scientific, Waltham, MA) and Syto 9 dye (Thermo Fisher Scientific) on the LSR II. Bacteria were frozen as dry cell pellets until reconstituted in PBS for use in stimulations. For stimulations, bacteria were added at 2.5 bacteria per leukocyte to 100 μl of whole blood or 500,000 isolated neutrophils in 1 ml R10 media (RPMI 1640 with 2.05mM L-glutamate, 100U/ml Penicillin, 100μg/ml Streptomycin, and 10% fetal bovine serum [all from GE Healthcare, Logan, UT]) and incubated aerobically for 20 hours at 37°C. Following the incubation, the supernatant was removed and the blood and isolated neutrophils were washed before being analyzed by flow cytometry.

### Statistical analyses

Differences in neutrophil frequencies and active Caspase-3 expression between infected and uninfected individuals were determined by Mann-Whitney test. In an effort to control for imbalances in potential confounders they were included as covariates in subsequent multivariate models. Adjusted multivariate analyses were conducted by regressing log-transformed neutrophil frequencies and active Caspase-3 expression on infection status, age, race (white vs. non-white), sex and sexual orientation. Note that sex and sexual orientation were treated as a single trinary variable, coded using two dummy variables, with female sex as the referent category. A paired one-way ANOVA was used to assess differences between groups stimulated with different bacteria or TLR agonists in the *in vitro* experiments followed by a Dunnett’s or Tukey’s post-hoc analysis for multiple comparisons. *p*-values reported are adjusted *p*-values from the post-hoc analysis. Correlations were assessed using the Spearman’s rank correlation analysis. Taxon counts at the family or genus level were center-log ratio (CLR)[74] transformed to accommodate compositionality and encourage normality. Taxa present in fewer than 25% of samples were omitted. Community level (beta-diversity) analyses were conducted using MiRKAT[75], which is a generalization of PERMANOVA[76], under Euclidean distance. For the adjusted analyses of associations with total neutrophil and neutrophil survival we included age, race, sex/sexual preference, and HIV status as covariates. For the adjusted analyses of associations with HIV status, we included age, race and sex/sexual preference as covariates. For the adjusted analyses based on MSM status we included age, race, and HIV status. Associations with individual taxa were determined by regressing the abundance of each taxon on the variable of interest and additional covariates as described above followed by false discovery rate[77] control for multiple testing.

In addition to community level and individual taxon level analyses, we used the previously published *selbal* approach [78] to explore whether balances of particular bacterial taxa could predict neutrophil survival. We considered the percentage of active Caspase-3 low, CD16 high neutrophils as the outcome and used the s*elbal* procedure as described to search for a global balance of taxa related to the outcome. No covariates were included.

## Supporting information

S1 Supporting Information Supplementary figures and tables. (DOCX)

S1 Supporting Data Flow cytometry data. (XLSX)

S2 Supporting Data Associations between individual taxa and total neutrophils and associations between individual taxa and neutrophil survival. (XLSX)
