# Supplementary material for: Increased mucosal neutrophil survival is associated with altered microbiota in HIV infection
Source: PLoS Pathog. 2019 Apr 11;15(4):e1007672. doi: 10.1371/journal.ppat.1007672 (PMC6459500; doi:10.1371/journal.ppat.1007672)

**Supporting Information:**


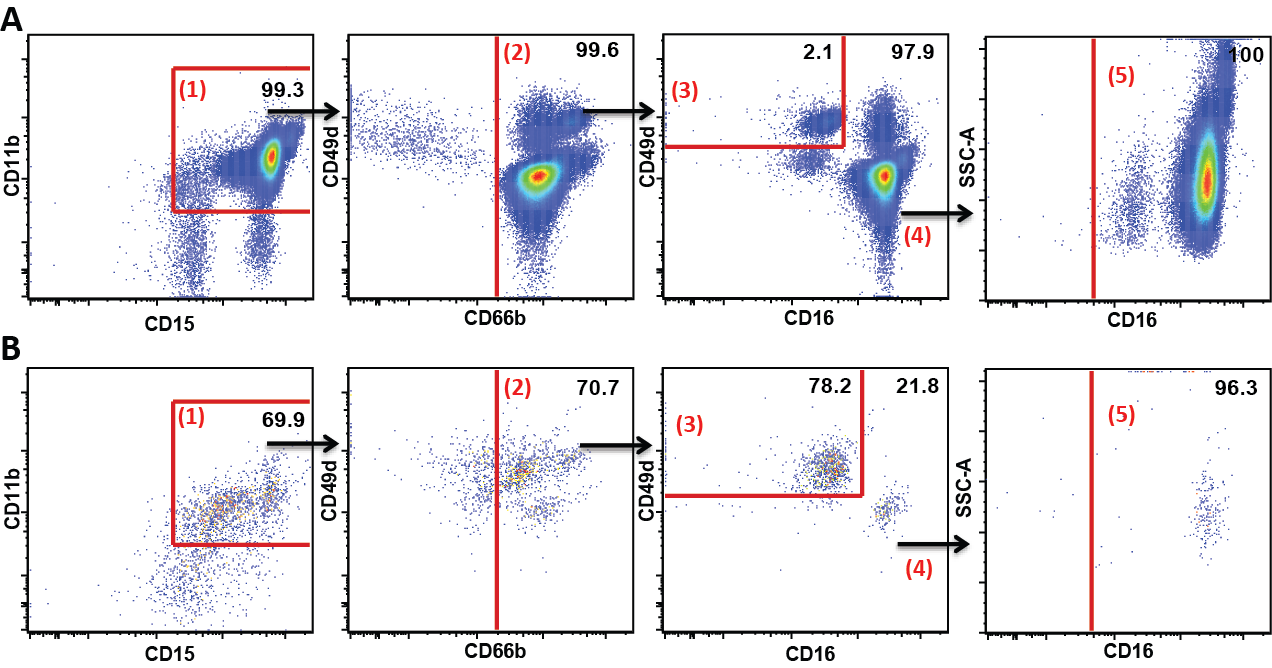


**Supplementary Figure 1. Representative flow cytometry gating of blood and GI neutrophils.** Representative plots showing live CD45+CD14-CD3-CD20-HighSSC cells in blood (A) and GI leukocytes (B) from an HIV+ individual further gated to identify neutrophils. Neutrophils and eosinophils are defined as CD11b+CD15+ (1) and CD66b+ (2). Eosinophils are then distinguished as CD49dhighCD16low (3) and a second overlapping exclusion gate captures the remaining cells (4). Non-eosinophils (4) are then gated for CD16+ neutrophils (5).


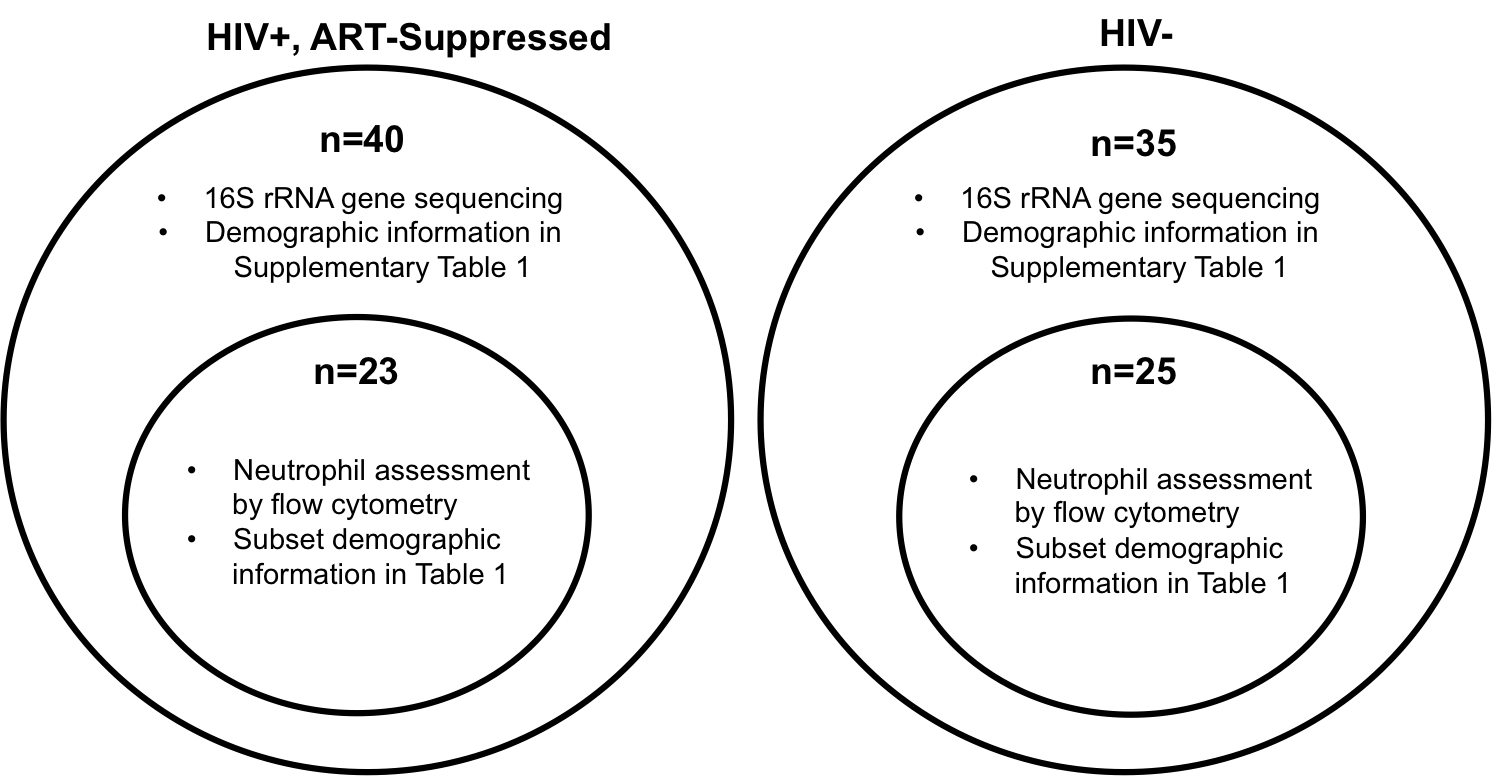


**Supplementary Figure 2. Schematic depicting colorectal biopsy samples collected and experimental assessments done on sample subsets.** Colorectal biopsies collected from all individuals were assessed for microbiome composition by 16S rRNA gene sequencing. A subset within that same cohort was assessed for neutrophil frequency and lifespan by flow cytometry due to constraints on real-time neutrophil assessments.

**
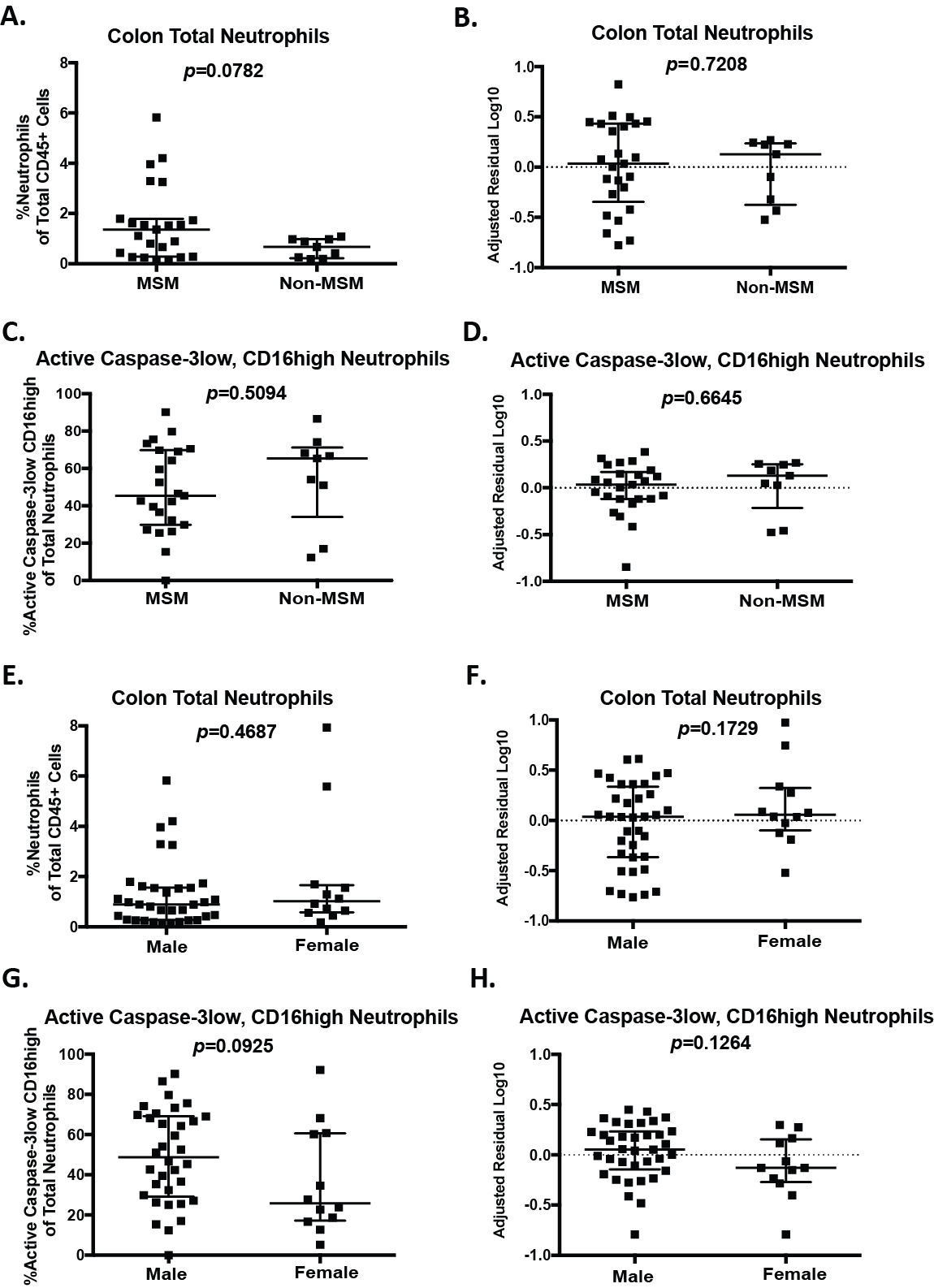
**

**Supplementary Figure 3. No effect of sexual orientation or sex on neutrophils.** A.) The frequency of neutrophils as a percentage of total leukocytes isolated from colorectal biopsies as measured by flow cytometry and compared based on sexual orientation. Includes data from male HIV-infected ART-suppressed individuals and male uninfected controls B.) Depiction of the log_10_ residuals of data presented in (A) from a multivariate analysis adjusted for age, race, and HIV status. C.) The frequency of total neutrophils expressing low levels of active Caspase-3 and high levels of CD16 in colorectal biopsies as measured by flow cytometry and compared based on sexual orientation. Includes data from male HIV-infected ART-suppressed individuals and male uninfected controls D.) Depiction of the log_10_ residuals of data presented in (C) from a multivariate analysis adjusted for age, race, and HIV status. E.) The frequency of neutrophils as a percentage of total leukocytes isolated from colorectal biopsies as measured by flow cytometry and compared based on sex. Includes data from HIV-infected ART-suppressed individuals and uninfected controls. F.) Depiction of the log_10_ residuals of data presented in (E) from a multivariate analysis adjusted for HIV status. G) The frequency of total neutrophils expressing low levels of active Caspase-3 and high levels of CD16 in colorectal biopsies as measured by flow cytometry and compared based on sex. Includes data from HIV-infected ART-suppressed individuals and uninfected controls. H.) Depiction of the log_10_ residuals of data presented in (G) from a multivariate analysis adjusted for HIV status. Statistical differences in neutrophil frequencies between infected and uninfected individuals were determined by Mann-Whitney test followed by a multivariate regression analysis.

**
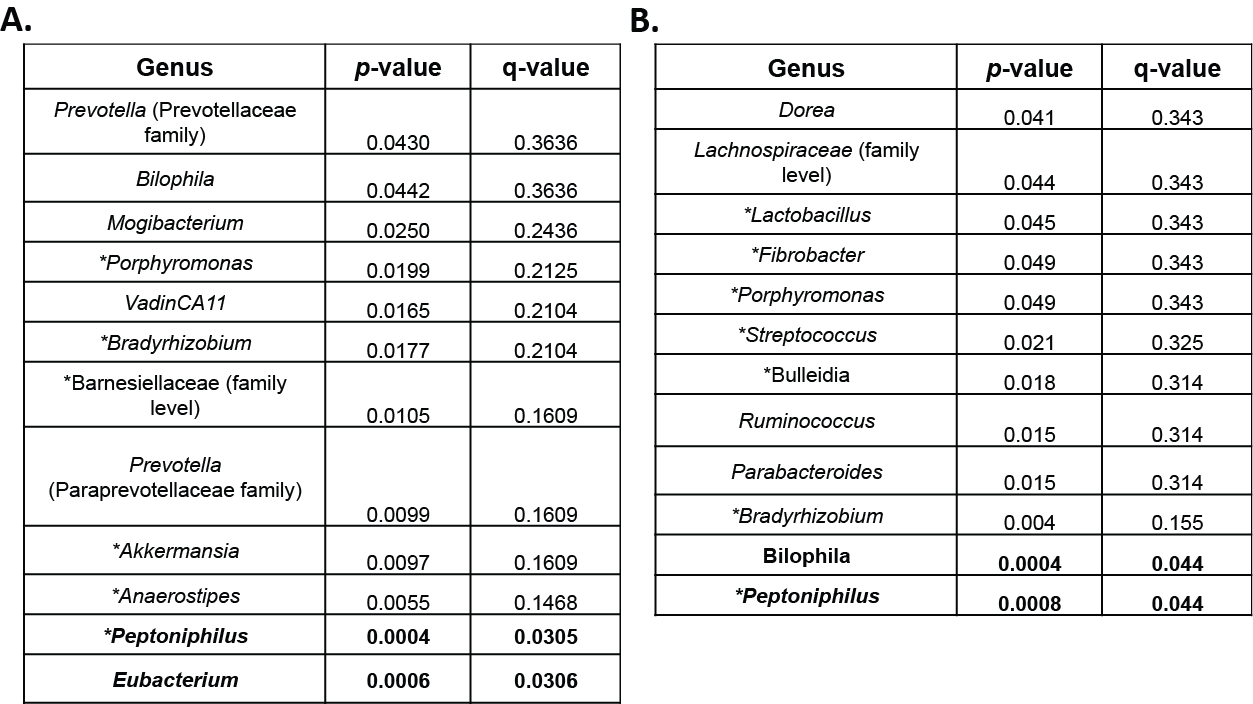
**

**Supplementary Figure 4. Genera associating with HIV status of colorectal biopsies.** Associations between individual genera as measured by 16S rRNA sequencing of colorectal biopsies and HIV status of HIV-infected, ART-suppressed and uninfected individuals. A.) The top genera associating with HIV status. B.)The top genera associating with HIV status following adjustment for age, race, sex, and sexual orientation. Individual associations between genera and HIV status were assessed by regressing genus abundance on HIV status and covariates with a q-value of <0.05 considered significant (**bolded** genera). Genera negatively associated with HIV status are indicated by *.


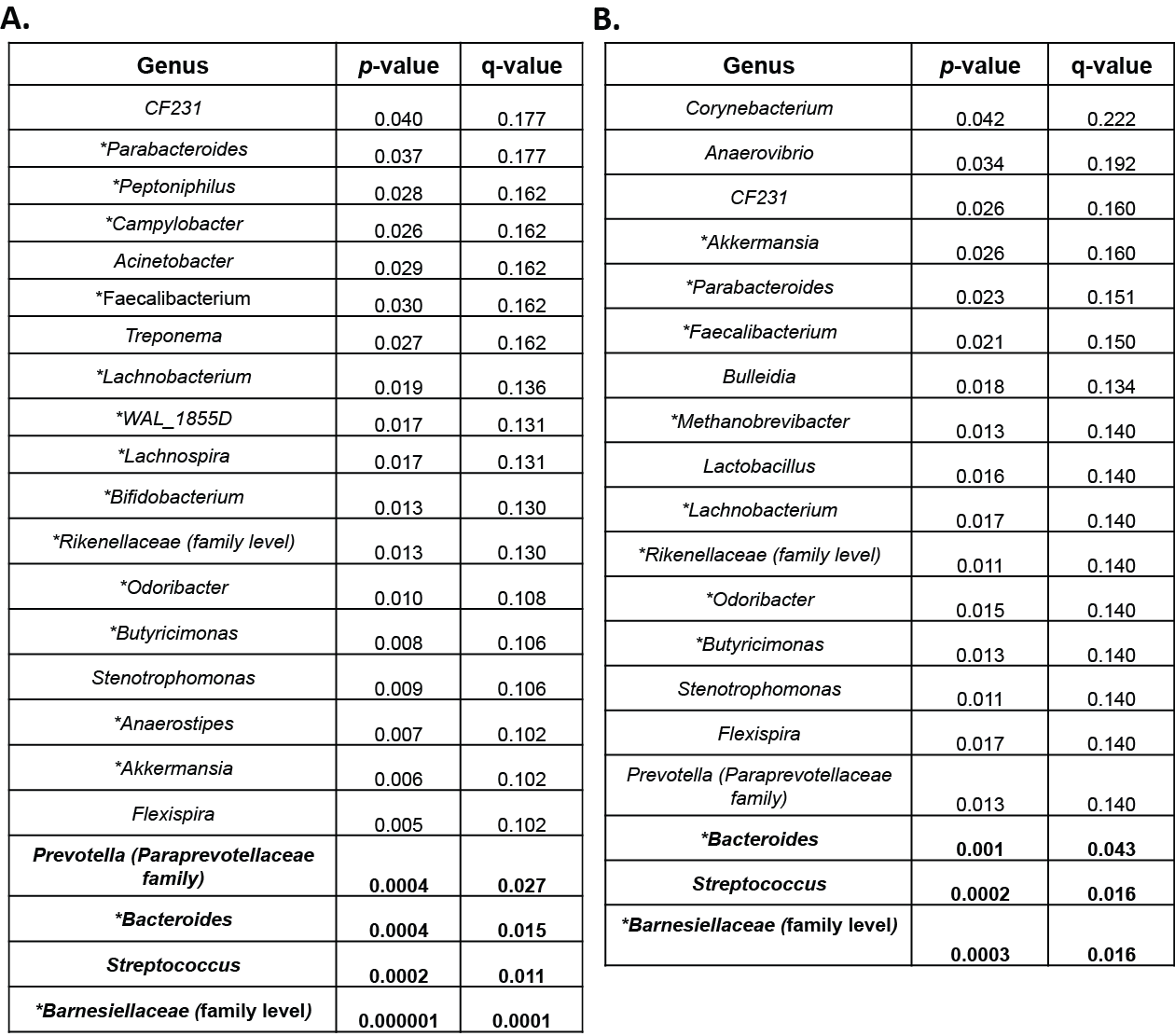


**Supplementary Figure 5. Genera associating with MSM sexual orientation of colorectal biopsies in men.** Associations between individual genera as measured by 16S rRNA sequencing of colorectal biopsies and sexual orientation of HIV-infected, ART-suppressed and uninfected men. A.) The top genera associating with MSM status. B.)The top genera associating with MSM status following adjustment for age, race, sex, and HIV status. Individual associations between genera and MSM status were assessed by regressing genus abundance on MSM status and covariates with a q-value of <0.05 considered significant (**bolded** genera). Genera negatively associated with MSM status are indicated by *.


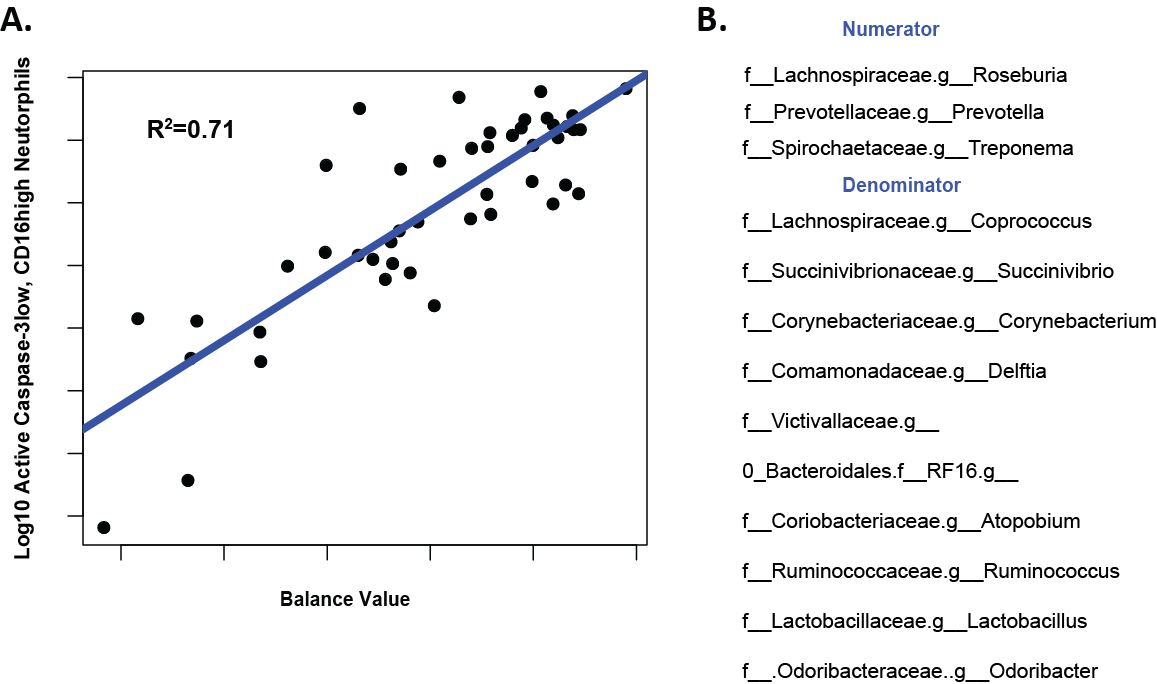


**Supplementary Figure 6.** Description of the microbiome global balance predictive of Active Caspase-3low CD16high Neutrophils. A. The regression model with Active Caspase-3Low CD16High neutrophils as a percentage of total neutrophils on the y axis and the balance value on the x axis. B. The two groups of taxa that form the global balance.

**
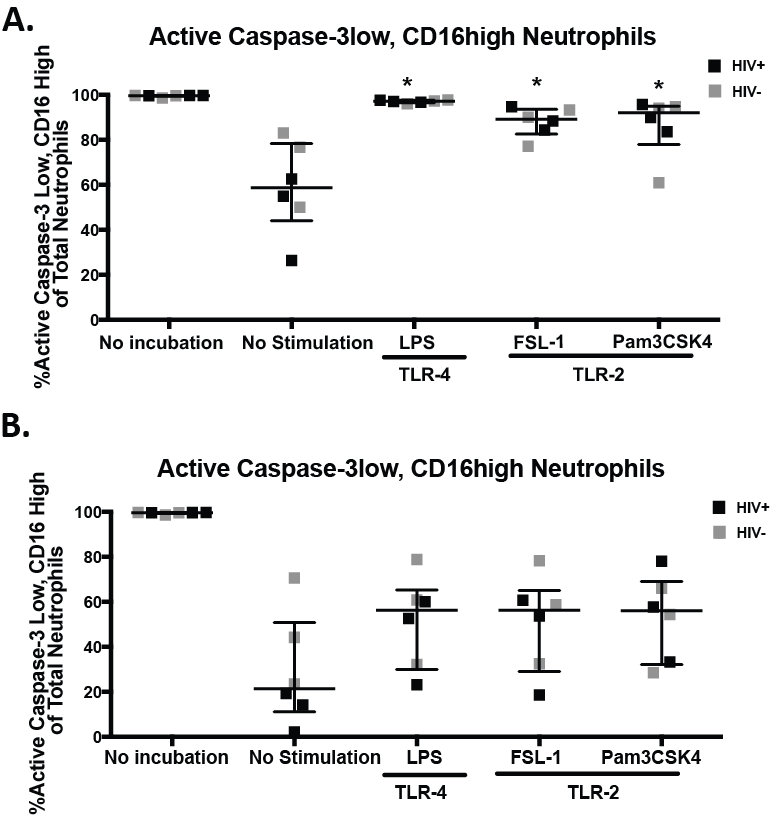
**

**Supplementary Figure 7. TLR-4 and TLR-2 stimulation decrease neutrophil apoptosis.** A.) Percentage of active Caspase-3low, CD16high neutrophils in whole blood incubated with TLR-4 and TLR-2 agonists as measured by flow cytometry. B.) Percentage of active Caspase-3low, CD16high neutrophils measured by flow cytometry after isolation with CD15+ magnetic beads followed by incubation with TLR-4 and TLR-2 agonists. Statistical significance was determined using a paired one-way ANOVA followed by a Dunnett’s post-hoc analysis for multiple comparisons comparing each group to the media control. Asterisks (*) indicate significance by adjusted *p*-value from post-hoc analysis (**p* <0.05).

**
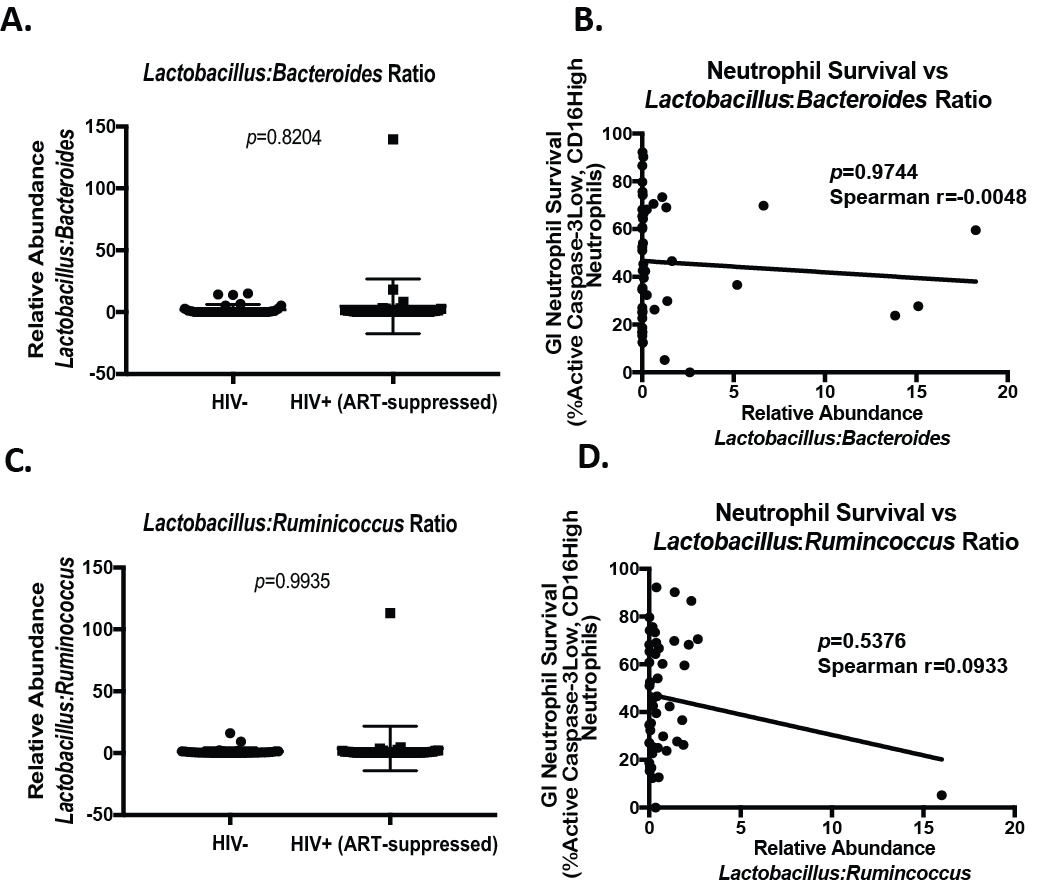
**

**Supplementary Figure 8. No associations between *Lactobacillus:Bacteroides* and *Lactobacillus:Rumincoccus* ratios and neutrophil survival.** A.) Ratio of *Lactobacillus:Bacteroides* (relative abundances) in HIV-negative and HIV-positive, ART-suppressed individuals. B.) Correlation of *Lactobacillus:Bacteroides* ratio and GI neutrophil survival as measured by the percentage of active Caspase-3 low, CD16 high neutrophils isolated from colorectal biopsies of HIV-negative and HIV-positive, ART-suppressed individuals. C.) Ratio of *Lactobacillus:Ruminococcus* (relative abundances) in HIV-negative and HIV-positive, ART-suppressed individuals. D.) Correlation of *Lactobacillus:Ruminococcus* ratio and GI neutrophil survival as measured by the percentage of active Caspase-3 low, CD16 high neutrophils isolated from colorectal biopsies of HIV-negative and HIV-positive, ART-suppressed individuals. Statistical differences between infected and uninfected individuals (A and C) were assessed by Mann-Whitney test with a *p-*value<0.05 considered significant. Correlations (B and D) were assessed by Spearman rank correlation test.


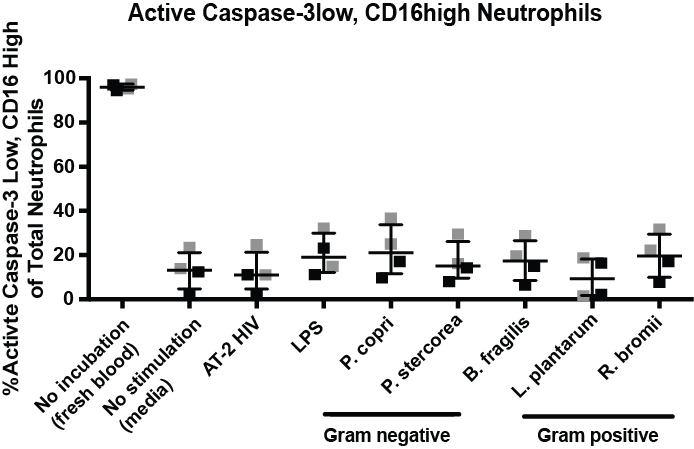


**Supplementary Figure 9. Reduced effect of bacteria on apoptosis of isolated neutrophils.** Percentage of active Caspase-3low, CD16high neutrophils measured by flow cytometry after isolation with CD15+ magnetic beads followed by incubation with different HIV altered mucosal bacteria. Statistical significance was assessed using a paired one-way ANOVA followed by a Dunnett’s post-hoc analysis for multiple comparisons comparing each group to the media control. No significant differences were identified.

**Supplementary Table 1. Study Participant Demographics (including individuals with no flow cytometry data)**


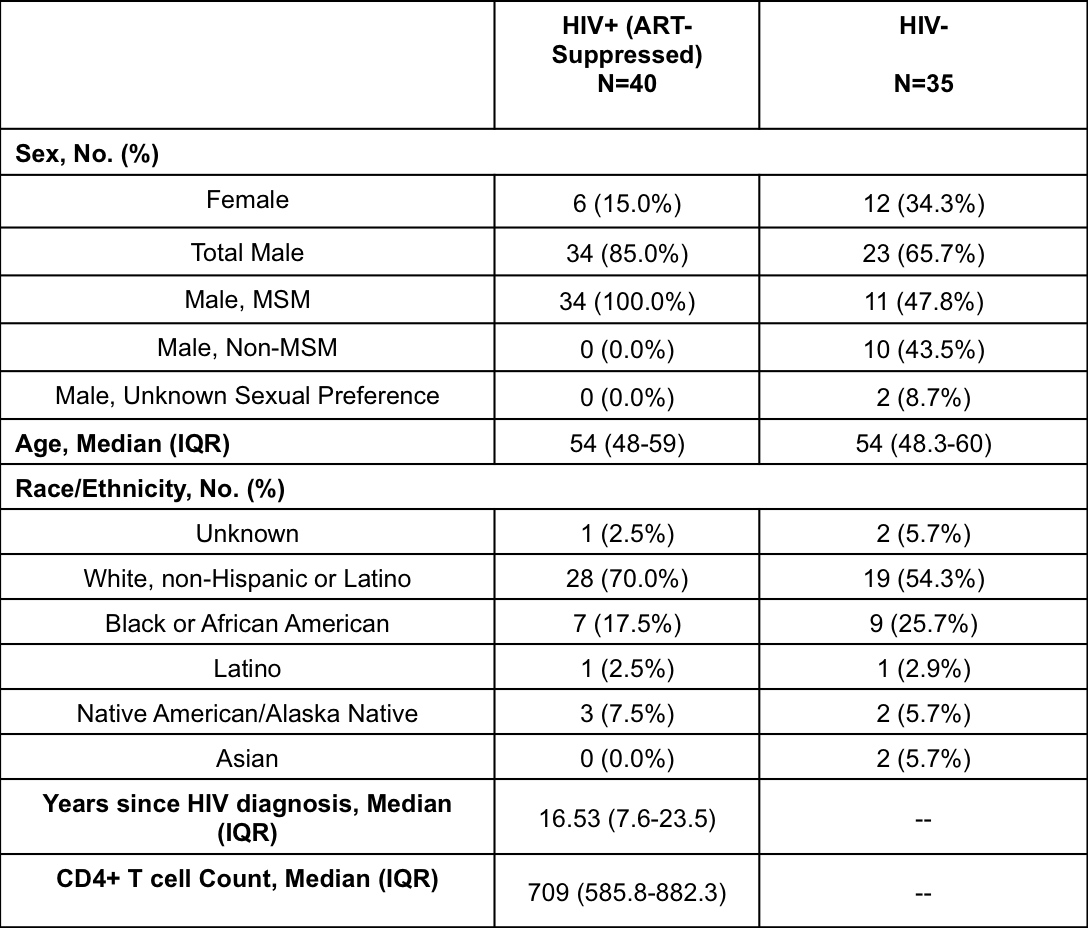

Supplement: S1 Supporting Information — (DOCX) [file ppat.1007672.s001.docx]
